# Weaker Dental Enamel Explains Dental Decay

**DOI:** 10.1371/journal.pone.0124236

**Published:** 2015-04-17

**Authors:** Alexandre R. Vieira, Carolyn W. Gibson, Kathleen Deeley, Hui Xue, Yong Li

**Affiliations:** 1 Department of Oral Biology, School of Dental Medicine, University of Pittsburgh, Pittsburgh, Pennsylvania, United States of America; 2 Department of Anatomy and Cell Biology, School of Dental Medicine, University of Pennsylvania, Pennsylvania, United States of America; 3 Department of Orthodontics, School of Stomatology, Fourth Military Medical University, Xi’an, PR China; Oregon Health and Science University, UNITED STATES

## Abstract

Dental caries continues to be the most prevalent bacteria-mediated non-contagious disease of humankind. Dental professionals assert the disease can be explained by poor oral hygiene and a diet rich in sugars but this does not account for caries free individuals exposed to the same risk factors. In order to test the hypothesis that amount of amelogenin during enamel development can influence caries susceptibility, we generated multiple strains of mice with varying levels of available amelogenin during dental development. Mechanical tests showed that dental enamel developed with less amelogenin is “weaker” while the dental enamel of animals over-expressing amelogenin appears to be more resistant to acid dissolution.

## Introduction

Dental caries is a leading cause of tooth loss in both developed and developing countries [1]. The disease affects billions of people and occasionally leads to lethality in both children and adults or important sequela, such as blindness [2–7]. To treat dental caries in the permanent dentition of children from developing countries by traditional amalgam restorative dentistry would require financial resources beyond the total health budget of these countries [8]. Children with poorer oral health status are more likely to experience dental pain, miss school, and perform poorly in school [9]. The aesthetic nature of untreated dental decay can at least indirectly compromise the child’s self-esteem and social development [10]. Vaccine discovery efforts have focused on the bacterial species associated with dental caries, particularly *Streptococcus mutans*. Difficulties developing a vaccine against caries have included serological cross-reactivity between the heart tissue antigens and certain antigens from haemolytic *Streptococci* in some patients with rheumatic fever and lack of effectiveness of oral administrations. Intranasal route targets have been explored as well as alternative target antigens [11].

A proverbial belief among populations is that some people are born with teeth that are less resistant to dental caries (“weak teeth”), while others are born with teeth that are more resistant to dental caries (“strong teeth”). This difference is attributed to things as diverse as excessive milk consumption to infections and prolonged and/or repetitive use of antibiotics during childhood. Dentists usually disregard those beliefs in favor of the assumption that good oral hygiene, limited use of carbohydrates between meals, and fluoride exposure would overcome any inherited weakness of teeth related to dental caries. Despite these assumptions, a subset of the population remains caries free, regardless of oral hygiene practices, fluoride exposure, and dietary habits. Conversely, individuals that report frequent tooth brushing, moderate ingestion of sugars and use of fluoridated toothpastes may have dental caries experience [12].

At the population level, dental caries remains a public health issue, and new strategies to prevent the disease are needed since fluoridation of drinking water and toothpastes cannot protect everyone [13] and vaccine development holds little promise at this time. One approach is to identify biological mechanisms that can be targeted by novel preventive strategies and aid the identification of individuals at higher risk to the disease.

The earliest studies of inheritance patterns in twins [14,15], families [16], and animal breeding [17] were consistent with a genetic component for susceptibility to dental caries. One underlying mechanism that could explain a genetic contribution to dental caries is the formation of dental enamel that is more susceptible to demineralization. Previous research by others and us has identified genetic variants in enamel formation genes that are associated with higher levels of caries experience [18–25]. One of these genes is the X-linked amelogenin (*AMELX*), which when mutated causes a disorder of tooth enamel called amelogenesis imperfecta [26]. These studies however are not easy to interpret; hence our focus here is on the level of *AMELX* expression rather than *AMELX* variants. Regarding dental caries, our hypothesis is that variable expression of amelogenin would result in enamel alterations that could make individuals more or less susceptible to acid demineralization and caries progression. We used twelve strains of transgenic mice that express variable levels of amelogenin to determine if reduced amelogenin levels would lead to “weaker” enamel and hence higher caries susceptibility.

## Methods

### Animals

Procedures were approved by the University of Pittsburgh and University of Pennsylvania Institutional Animal Care and Use Committee (IACUC 804584), and follow ARRIVE guidelines. For this study, anaesthesia was not required, as the study did not involve surgical intervention. Sacrifice was by carbon dioxide administration.

For the *Amelx* models, breeding pairs were maintained with the appropriate genetic background for null, transgenic and wild type mice. Each mouse was genotyped using high molecular weight DNA isolated from mouse tails for transgene status and genetic background where necessary by PCR using *Amelx* oligomer primers as described [27]

Matings within or between strains produced postnatal day 4 (PN4), 6-week, or 5-month mice for the experiments described. Males and females were used. Mice (n = 108) were sacrificed by administration of CO_2_ and the mandibles containing incisors and molar teeth were dissected from transgenic and wild-type (WT) mice.

Mice were generated that lack amelogenin (*Amelx*KO) [28] or express a transgene that encodes the most abundant Amelx protein M180 [27] were compared to WT. The KO and transgenic strains have been moved to the caries-susceptible C57BL/6J genetic background [29] by repeated mating. Since C3H/HeJ mice are caries-resistant [29], we generated mixed B6/C3H/HeJ strains that were *Amelx* null by repeated matings until N5 was reached, using approximately 58 mice with genotyping of tail DNA by PCR. Teeth in dissected C3HKO(N5) mandibles were examined by micro-indentation and compared to B6KO and WT mice (total = 238 mice).*Amelx* heterozygous females with the two backgrounds were also compared.

The *Amelx* KO mutation was transferred from C57BL/6J to C3H/HeJ

**Fig 1:**
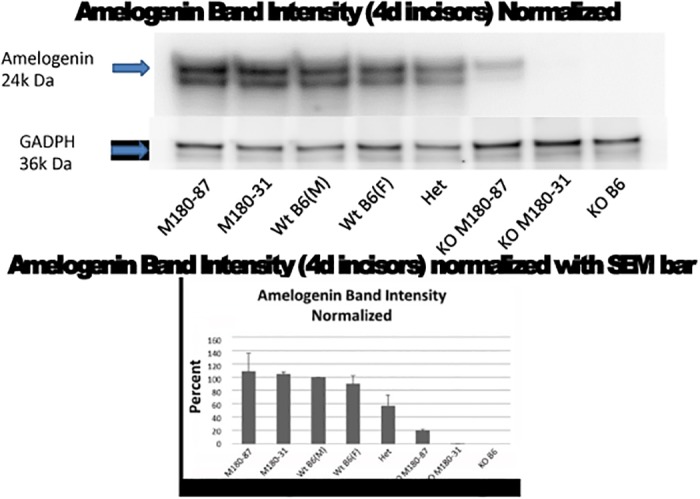
Mouse strains and Amelx expression levels. Western blot analysis confirms predictions from Table 1.

Transgenic mice on a wild-type (WT) background are expected to have more amelogenin protein compared to WT, and heterozygous (*Amelx* +/-) females should have approximately half the WT amount (Fig 1). KO and transgenic mice were mated and transgene+/KO mice expressed a single amelogenin gene [30].

### Western Blots

Mice of each genotype (n = 6) at PN4 were sacrificed and extracts were prepared from molars and incisors in order to estimate the amount of Amelx protein by Western blot using an Amelx antibody (Santa Cruz Biotechnology, Inc., Santa Cruz, CA), as has been done for several of these strains previously [27,30]. The relative amounts of Amelx were analyzed by ImageJ software in order to confirm predictions in Table 1 (Fig 1).

**Table 1:** Predicted relative amount of Amelx protein in various murine Amelx transgenic and null strains.

Proposed Amelogenin Expression	Genotype	First described in:	AMELX expression described:
0	KO/B6		
0	KO/mix	[28]	[28]
Least	TgM180-31/KO ♂	[27]	[27,30]
	TgM180-87/KO ♂	[30]	
	*AMELX* het ♀		[27]
	WT		[30]
	TgM180-31	[27]	
Most	TgM180-87	[38]	

### Dental Enamel Microhardness Tests

Samples from each strain at 6 weeks and 5 months (N = 6) were tested for enamel mechanical hardness. Samples were stored in a moist environment in the presence of 0.1% thymol to retard bacterial growth [31].

Since enamel is a brittle material, the Knoop hardness test was indicated. In this test, only a small indentation is created by a pyramidal diamond point, which is pressed into the polished enamel surface with a known force, for a specific dwell time. The resulting indentation is measured using a microscope. The Knoop hardness number KHN is the ratio of the load applied to the indenter, P (kgf) to the unrecovered projected area A (mm^2^): KHN = F/A = P/CL^2^. Where: F = applied load in kgf; A = the unrecovered projected are of the indentation in mm^2^; L = measured length of long diagonal of indentation in mm; C = 0.07028 = Constant of indenter relating projected area of the indentation to the square of the length of the long diagonal.

Experiments were done perpendicularly to the external surface of the enamel [22]. At the start of sample preparation the enamel surface were polished to obtain a flat surface. Blocks were then submitted to baseline microhardness analysis using a microhardness tester (IndentaMet 1100 Series, Buehler Ltd., Lake Bluff, IL, USA) with a knoop diamond under a load of 25 grams for 5 seconds. One indentation was made.

### Artificial Caries Procedure

Young adult (6 weeks; n = 60) and older mice (5 months; n = 108) were sacrificed and mandibles prepared for analysis by the caries protocol described for the micro-indentation assays. Artificial caries lesions were created by immersing each enamel block in 24 mL of demineralizing solution (1.3 mmol/L Ca, 0.78 mmol/L P, 0.05 mol/L acetate buffer, 0.03 μgF/mL, pH 5.0) at 37°C for 16 hours. This method produces a subsurface enamel demineralization without surface erosion [32–35]. Surface microhardness was measured again by another indentation created underneath the initial one.

### Statistics

The baseline microhardness and rates of change of microhardness scores after artificial caries creation were calculated. ANOVA was used to determine statistical significant differences with alpha at 0.05.

## Results

Our hypothesis was that enamel samples formed under conditions of lower Amelx gene expression would be more susceptible to caries formation. Because Amelx proteins resulting from alternative splicing of the primary RNA transcript have varying expression levels, the models could be compared according to relative amounts of amelogenin protein expressed during enamel development (Table 1). Fig 1 indicates that the amounts of Amelx in each strain did vary as expected, as shown by Western blot of teeth probed with an Amelx antibody. More than one Amelx band is visible as the C-terminus is cleaved almost immediately after secretion.

**Fig 2:**
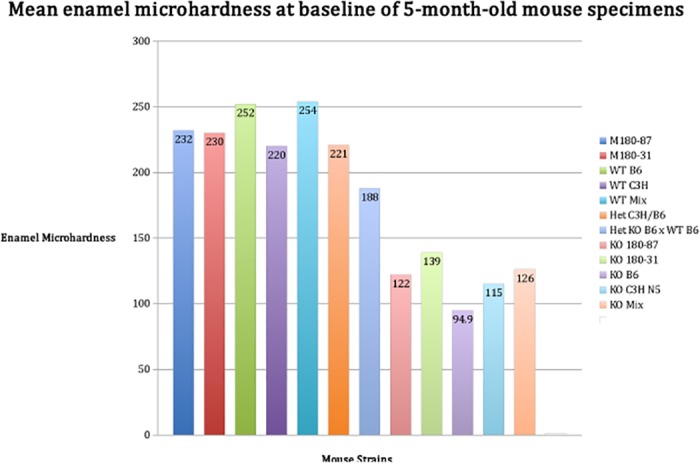
Mean enamel microhardness of 5-month old mice developed with variable amounts of amelogenin. Knockout mice (KO B6, KO C3H N5, KO Mix), which express no Amelx have “softer” enamel in comparison to the other strains (ANOVA; p<0.0001).

We tested the samples from males and females at baseline and after exposure to an artificial caries protocol with the goal of defining if samples from strains with different levels of Amelx protein are differently influenced by high caries challenge conditions [32–35]. Enamel microhardness results showed that low AMELX levels led to weaker enamel (Fig 2, ANOVA; p < 0.0001).

**Fig 3:**
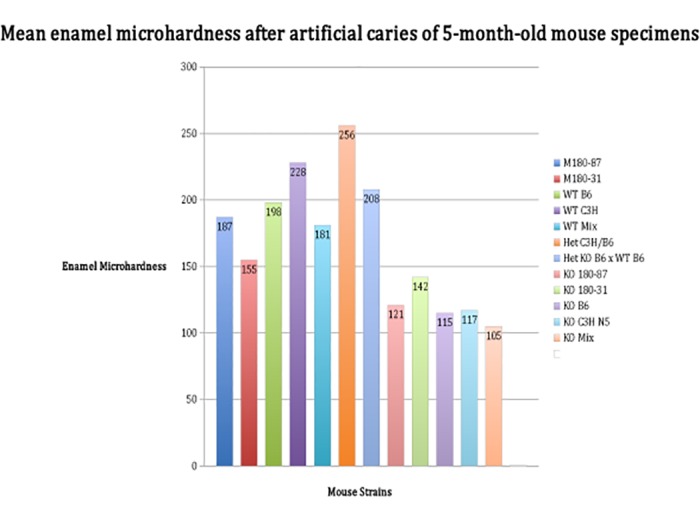
Mean enamel microhardness of 5-month old mice developed with variable amounts of amelogenin after the creation of an artificial caries lesion. The transgenic line Het C3H/B6 is more resistant to acid demineralization in comparison to knockout mice (KO B6, KO C3H N5, KO Mix) (ANOVA; p<0.0001).

After the creation of an artificial caries lesion, the pattern of enamel microhardness among mouse strains is similar with levels being lower in comparison to baseline values (Fig 3). The values indicate that the dental enamel shows initial stages of demineralization. These results are similar on samples from 6-week mice (data not shown).

## Discussion

Our study shows that lower levels of AMELX protein during enamel development predisposes to dental caries because enamel is “weaker” or “softer” to begin with. Also, excess AMELX creates harder dental enamel that is more resistant to acid demineralization.

These results provide a rationale for association studies that provide evidence that genetic variation in genes involved in enamel formation is associated with high caries experience [18–25]. The main limitation of these association studies is that they did not provide data that support a mechanism to explain the results. A very plausible mechanism is that genetic variation will influence the formation of the dental enamel and make it more or less susceptible to demineralization under acidic conditions, which is relevant for the pathogenesis of dental caries and enamel erosion.

The discovery that AMELX level differences during dental enamel development will translate into “weaker” teeth provides a venue for new preventive strategies for dental caries. Individuals with specific amelogenin genetic variants may be recommended for more frequent preventive therapies such as highly concentrated professional fluoride applications. The utilization of dental sealants on occlusal surfaces of molars [36] and resin infiltration [37] to “coat” proximal surfaces of posterior teeth may be recommended for individuals considered at higher risk for dental caries based on their amelogenin genetic profiles. Dental caries still affects disproportionally the poor and disenfranchised, which in the United States are over-represented by underserved minorities. Since the associated amelogenin single nucleotide polymorphism rs946252 [22] has the less common allele frequency varying from 13% to 17% in African Americans and individuals with Mexican ancestry (according to the 1000 Genomes browser), it could be used to define individuals that may benefit from more aggressive dental caries preventive measures (*i*.*e*., dental sealants).
